# Fabrication and Evaluation of Water Hyacinth Cellulose-Composited Hydrogel Containing Quercetin for Topical Antibacterial Applications

**DOI:** 10.3390/gels8120767

**Published:** 2022-11-24

**Authors:** Tanpong Chaiwarit, Baramee Chanabodeechalermrung, Nutthapong Kantrong, Chuda Chittasupho, Pensak Jantrawut

**Affiliations:** 1 Department of Pharmaceutical Sciences, Faculty of Pharmacy, Chiang Mai University, Chiang Mai 50200, Thailand; 2 Department of Restorative Dentistry, Faculty of Dentistry, Khon Kaen University, Khon Kaen 40002, Thailand; 3 School of Dentistry, Mae Fah Luang University, Chiang Rai 57100, Thailand; 4 Cluster of Research and Development of Pharmaceutical and Natural Products Innovation for Human or Animal, Chiang Mai University, Chiang Mai 50200, Thailand

**Keywords:** water hyacinth, cellulose, hydrogel, quercetin, antibacterial

## Abstract

Water hyacinth is an aquatic weed species that grows rapidly. In particular, it causes negative impacts on the aquatic environment and ecological system. However, water hyacinth is rich in cellulose, which is a biodegradable material. This study isolated cellulose from the water hyacinth petiole. It was then used to fabricate composite hydrogels made with water hyacinth cellulose (C), alginate (A), and pectin (P) at different mass ratios. The selected water hyacinth cellulose-based hydrogel was incorporated with quercetin, and its properties were evaluated. The FTIR and XRD of extracted water hyacinth cellulose indicated specific characteristics of cellulose. The hydrogel which consisted of the water hyacinth cellulose alginate characterized pectin: pectin had a mass ratio of 2.5:0.5:0.5 (C_2.5_A_0.5_P_0.5_), showed good puncture strength (2.16 ± 0.14 N/mm^2^), the highest swelling index (173.28 ± 4.94%), and gel content (39.35 ± 0.53%). The FTIR showed an interaction between water hyacinth cellulose and quercetin with hydrogen bonding. The C_2.5_A_0.5_P_0.5_ hydrogel containing quercetin possessed 92.07 ± 5.77% of quercetin-loaded efficiency. It also exhibited good antibacterial activity against *S. aureus* and *P. aeruginosa* due to hydrogel properties, and no toxicity to human cells. This study indicated that water hyacinth cellulose-composited hydrogel is suitable for topical antibacterial applications.

## 1. Introduction

*Eichhornia crassipes* (Mart.) Solms has the common name “lilac devil” or “water hyacinth”. It is an invasive species and one of the most problematic aquatic weeds in the world [1], because it is resistant to changes in environmental conditions and can reproduce quickly. One water hyacinth plant may multiply up to 1000 times in one month. In addition, water hyacinth also causes problems in agriculture, irrigation, and public health. Water hyacinth is an obstacle to the drainage of weirs and drainage gates, impedes fish growth, and is a habitat for disease-carrying animals, such as Bithynia mollusks, which carry liver fluke disease, along with mosquito larvae. Water hyacinth also changes the environment of water resources. Removing water hyacinths can be achieved using machinery, chemical herbicides, or biological methods such as insects or plant diseases [2]. However, using these disposal methods is costly and causes negative impacts on water resource ecosystems, such as the destruction of beneficial aquatic plants and water pollution [2]. Therefore, utilizing water hyacinths for other purposes is an environmentally friendly option for reducing the number of water hyacinths. The stem of water hyacinths, called the “petiole”, contains a high cellulose content. It consists of 25% cellulose, 33% hemicellulose, and 10% lignin [2,3]. The chemical characterization of hyacinth petioles showed that they contain approximately 23–57% by weight of cellulose compared to the dry plant weight [4,5,6,7]. Hyacinths are easy to find and are used as a source of cellulose at almost no cost.

Cellulose is a natural polymer found in all plants, but it is found mainly in cotton, linen, wood, bamboo, sugarcane, corncobs, banana plants, aquatic weeds, and water hyacinths. Some studies have found that hyacinths contain high cellulose levels and a low percentage of lignin compared to rice, wood, bamboo, and sugarcane [8,9]. Some researchers have extracted cellulose from water hyacinths with slightly different methods and conditions [2,3,6]. Chemical isolation is a common method used to isolate cellulose from plant materials. Alkaline hydrolysis and bleaching are effective steps for isolating cellulose from hemicellulose and lignin [6]. In this study, cellulose was isolated according to the method of Setyaningsih et al. [2]. The isolation consisted of the following steps: bleaching, alkaline hydrolysis, second bleaching, and acid hydrolysis. Cellulose and its derivatives are widely used in pharmaceutics. They are readily available, compatible with the body, non-toxic, and biodegradable. Cellulose is used to produce some materials, such as biodegradable membranes, excipients, absorbents, and hydrogels [9].

Hydrogels are three-dimensional polymeric networks that can absorb large amounts of water or fluids. They consist of a hydrophilic polymer chain that can absorb and store large amounts of water in the arrangement between the internal polymer chains [10]. Hydrogels are widely used, especially in soft tissue engineering and wound dressing. The insoluble hydrophilic structure of a hydrogel can absorb wound secretions and allow oxygen to pass through, accelerating wound healing. Moreover, the three-dimensional structure of hydrogels can swell several times after contact with water or a fluid compared to their original state, allowing them to retain moisture in the area when used effectively [11,12]. In addition, hydrogels are responsible for protecting wounds and maintaining moisture. They can also enhance the therapeutic effect by storing antimicrobial agents, growth factors, and macromolecule biological agents, enabling them to perform various functions [13]. Hydrogels prepared from natural polymers may be called “biopolymer-based hydrogels”, which have advantages over synthetic polymer hydrogels in terms of compatibility with the body, biodegradability, non-toxicity, and the ability to mimic tissues [14]. Although several studies using cellulose from other sources, including bacterial cellulose, and cellulose derivatives in hydrogel compositions have been published, few studies have investigated hydrogels composed of native water hyacinth cellulose (WHC). WHC is successfully used to form hydrogels using glutaraldehyde as a cross-linking agent [2]. However, the covalent cross-linking, such as glutaraldehyde toxic residue, is a drawback due to unreacted covalent-crosslinking agents. To avoid this problem, this study fabricated a composited hydrogel from WHC, sodium alginate, and pectin. To form an ionic interaction between pectin and sodium alginate, CaCl_2_ (non-toxic cross-linker) was used.

**Figure 1:**
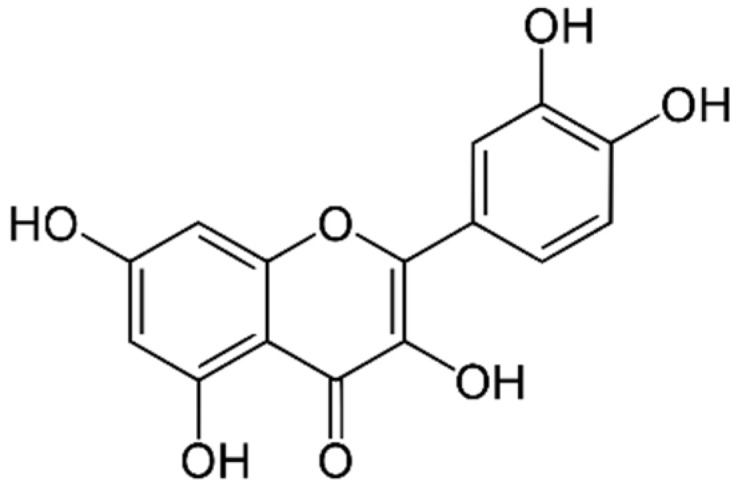
The chemical structure of quercetin.

Quercetin is a substance that belongs to the class of flavonoids and can be found in many plants. The chemical structure of quercetin is shown in Figure 1. It has various biological effects such as antioxidant effects against cancer cells, anti-inflammatory effects, and antimycobacterial effects including efficacy against pathogenic skin bacteria such as *Staphylococcus aureus* and *Pseudomonas aeruginosa* [15]. Some studies have developed quercetin-loaded hydrogels for wound healing applications from various polymers, such as polyvinyl alcohol (PVA) and gelatin/carrageenan [16,17]. However, the studies evaluating cellulose-composited hydrogels are limited. Therefore, this present study aimed to extract cellulose from water hyacinth, and the resulting extracted cellulose was used to prepare cellulose-composited hydrogels. Then, quercetin was incorporated into the selected cellulose-composited hydrogel as an active ingredient for wound dressing and antibacterial skin applications.

## 2. Results and Discussion

### 2.1. Water Hyacinth Cellulose Extraction

The cellulose was isolated from water hyacinth by dewaxing, bleaching, and alkaline hydrolysis. The dewaxing process can remove wax, oils, resin, organic compounds, pigments, and extractive compounds from the petiole of water hyacinth, but lignin, hemicellulose, and cellulose remain in the extracted material [3,9,18]. The dewaxed product was brown, indicating that other impurities in the water hyacinth fibers were not removed, such as lignin and hemicellulose. The bleaching process with 3% NaOCl not only increased the whiteness degree of the bleached sample but also eradicated lignin by breaking the ether bonds [3,19]. Alkaline hydrolysis with 1% NaOH can remove hemicellulose from the main chain, namely cellulose. Furthermore, the reaction with NaOH breaks down ester bonds connecting lignin with hemicellulose in a complex lignin–carbohydrate network, resulting in the dissolution of the lignin component [9,20]. A second bleaching with 1% NaOCl and acid hydrolysis with 5% HCl can remove the remaining lignin and hemicellulose [3]. In this study, the isolated product was white, indicating that other impurities including lignin and hemicellulose were removed [3]. The yield of the isolated product obtained from the isolation process was 19.24 ± 2.17% *w*/*w* of dry raw material. This extraction yield was possible and reasonable because the petiole of water hyacinth is composed of a high content of cellulose, at around 23–57% [4,5,6]. Compared with other studies using similar isolation processes, the extraction yield from Setyaningsih et al. [2] was only 8.95%, and our study’s yield was comparable with Sun et al. [21], exhibiting 19.38 ± 0.59% extraction yield. However, the variation in the composition of lignocellulosic compounds in water hyacinth depends on location, the part of plant materials, and the harvesting time [22]. These factors may lead to variations of extraction yield in each study.

### 2.2. Cellulose Identification

**Figure 2:**
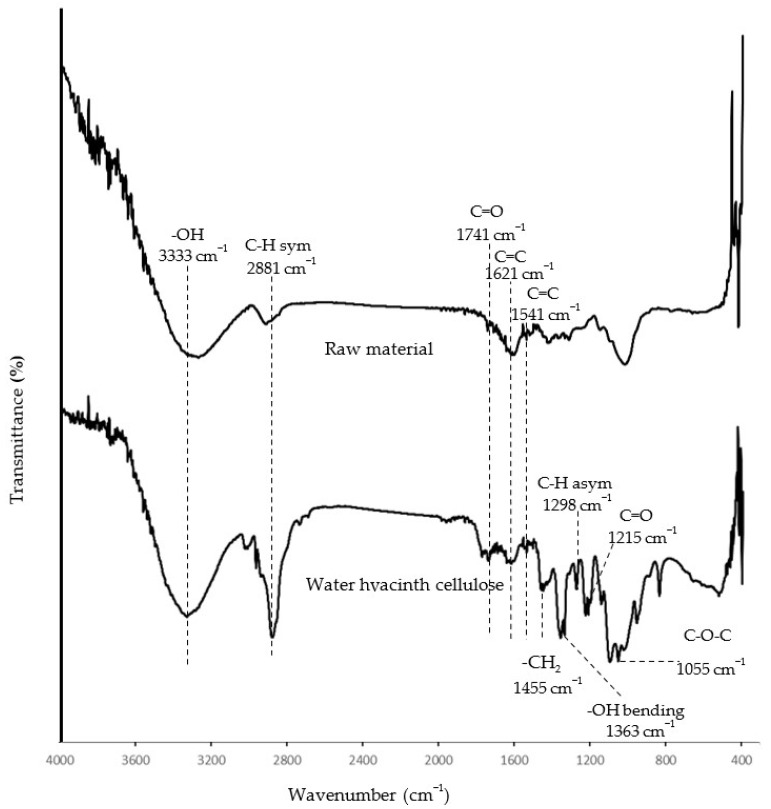
FTIR spectra of water hyacinth cellulose and raw material.

To ensure a high content of cellulose in the isolated product, the extracted matter was investigated using an FTIR spectrometer to identify the cellulose functional groups. Overall, the spectrum of raw material (petiole of water hyacinth) showed a similar pattern regarding the water hyacinth cellulose, but the extracted cellulose showed a clearer shape and more defined peaks when compared to the raw material (Figure 2). This indicated the isolated product was more purified and contained a relatively higher content of cellulose than the raw material. Thus, the isolated product could be referred to as water hyacinth cellulose (WHC). The FTIR spectra of the WHC in Figure 2 showed significant peaks at 3333 and 2881 cm^−1^, corresponding to the -OH stretching and C-H stretching, respectively. Moreover, the bands at 1455, 1363, 1298, and 1055 cm^−1^ relate to -CH_2_ scissoring, -OH bending, C-H asymmetric stretching, and the C-O-C pyranose ring, respectively. [23,24]. All the bands mentioned above correspond to the cellulose structure. However, the absorption bands at 1741 and 1215 cm^−1^ are associated with the C=O stretching group of acetyl group in hemicellulose. The peaks at 1621 and 1541 cm^−1^ relate to C=C aromatic ring stretching and C-H deformation in lignin, respectively [23]. These observations indicate that some hemicellulose and lignin remained in the extracted cellulose from the water hyacinth. This conclusion was possible and reasonable because several studies isolating cellulose from water hyacinth with alkaline hydrolysis and bleaching found that the isolated product consisted of hemicellulose and lignin as a minority [5,8]. For example, Tanpichai et al. [5] found that the obtained cellulose consisted of 82.5% *w*/*w* of cellulose, 4.1% *w*/*w* of hemicellulose, and 1.8% *w*/*w* of lignin. Pakutsah and Aht-Ong [8] found that the extracted cellulose was composed of cellulose (76.6% *w*/*w*), small amounts of hemicellulose (14.9% *w*/*w*), and lignin (1.6% *w*/*w*).

### 2.3. XRD Analysis

**Figure 3:**
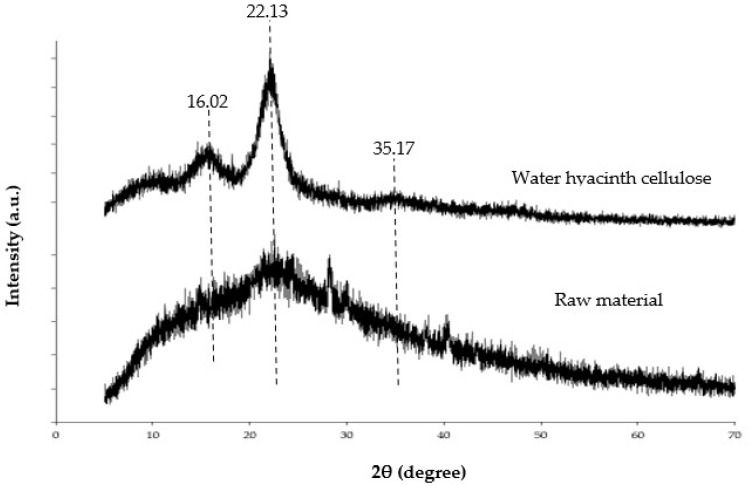
X-ray diffraction of water hyacinth cellulose and raw material.

XRD analysis is generally used to evaluate the material’s crystalline structure and degree of crystallinity [8]. The XRD pattern of water hyacinth cellulose revealed significant peaks around 16.02, 22.13, and 35.17 2θ related to its crystalline structure [2,8]. However, the XRD pattern of water hyacinth powder cannot clearly show the critical peaks due to the presence of amorphous forms of lignin and hemicellulose in the powder [8]. Thus, the elimination of lignin and hemicellulose escalates the crystallinity ratio and leads to sharper characteristic peaks of cellulose. This is shown in Figure 3 as the relative crystallinity of water hyacinth cellulose and water hyacinth powder. This experiment indicated that the extraction process had the potential to isolate cellulose from raw material. Thus, in this study, the extracted matter can be referred to as WHC. Moreover, the swelling properties of the hydrogel might be related to the crystallinity of cellulose, since a high crystallinity can decrease the water-holding capacity of a hydrogel [2].

### 2.4. Morphology and Properties of WHC-Composited Hydrogel

#### 2.4.1. Appearance of Water WHC-Composited Hydrogels

**Figure 4:**
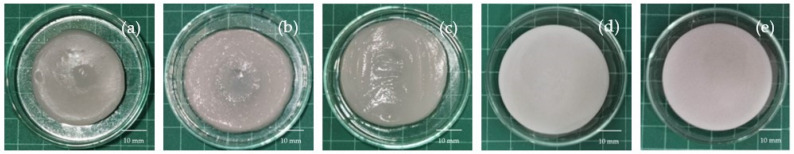
The appearance of WHC-composited hydrogels: C_1_A_0.5_P_0.5_ (**a**), C_1.5_A_0.5_P_0.5_ (**b**), C_2_A_0.5_P_0.5_ (**c**), C_2.5_A_0.5_P_0.5_ (**d**), and C_3_A_0.5_P_0.5_ (**e**).

The visual appearance of all hydrogel formulations is shown in Figure 4. All formulations of water hyacinth cellulose-based hydrogel (WHC-based hydrogel) were a circle with a white color. The C_1_A_0.5_P_0.5_, C_1.5_A_0.5_P_0.5_, and C_2_A_0.5_P_0.5_ hydrogels exhibited a collapse of the surface at the center of the hydrogels, possibly due to incomplete hydrogel formation. This result was similar to a previous study. Cellulose/sodium alginate hydrogel with a low ratio of cellulose was difficult to form because the network was too weak to hold substantial water [25]. These formulations also showed a larger standard deviation of thickness (Table 1) than C_2.5_A_0.5_P_0.5_ and C_3_A_0.5_P_0.5_. This phenomenon occurred due to the low ratio of cellulose in the formulations [26]. The thickness of WHC-composited hydrogels tended to increase with the addition of water hyacinth cellulose. Thus, C_3_A_0.5_P_0.5_ exhibited the highest thickness (5.71 ± 0.23 mm). On the other hand, the thinnest formulation was C_1_A_0.5_P_0.5_ (4.05 ± 0.71 mm). The diameter of WHC-composited hydrogels was around 40–44 mm, and C_1_A_0.5_P_0.5_ exhibited a significantly lower diameter (40.63 ± 3.13 mm) than C_2.5_A_0.5_P_0.5_ (44.75 ± 1.11) and C_3_A_0.5_P_0.5_ (44.43 ± 1.25 mm), respectively (*p* < 0.05).

#### 2.4.2. Morphology of WHC-Based Hydrogels

**Figure 5:**
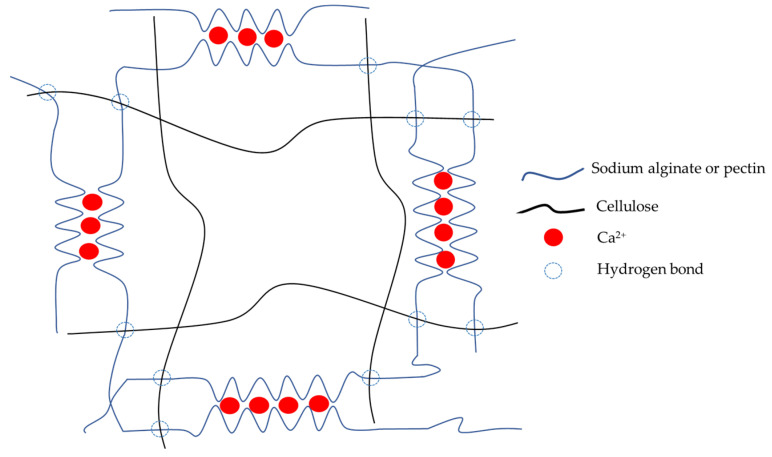
The illustrative scheme of hydrogel formation.

The scheme of the mechanism of hydrogel formation is shown in Figure 5. COO^−^ groups of sodium alginate and pectin interact with Ca^2+^ to form the primary structure of the hydrogel. Cellulose forms hydrogen bonds with pectin and the alginate polymer chain to enhance the stability and strength of the hydrogel.

**Figure 6:**
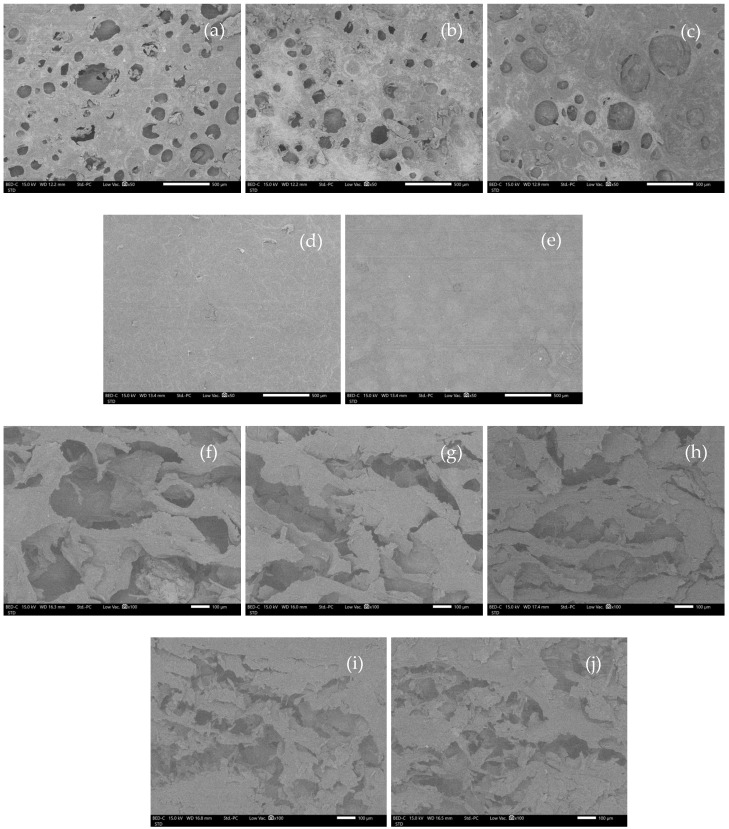
SEM micrographs of the surface and cross section of WHC-based cryogels at 50 and 100 magnifications. (**a**) = surface of C_1_A_0.5_P_0.5_, (**b**) = surface of C_1.5_A_0.5_P_0.5_, (**c**) = surface of C_2_A_0.5_P_0.5_, (**d**) = surface of C_2.5_A_0.5_P_0.5_, (**e**) = surface of C_3_A_0.5_P_0.5,_ (**f**) = cross-sectional image of C_1_A_0.5_P_0.5_, (**g**) = cross-sectional image of C_1.5_A_0.5_P_0.5_, (**h**) = cross-sectional image of C_2_A_0.5_P_0.5_, (**i**) = cross-sectional image of C_2.5_A_0.5_P_0.5_, and (**j**) = cross-sectional image of C_3_A_0.5_P_0.5_.

The appearance of WHC-based hydrogels after freeze-drying (cryogels) was similar to sponges after using visual examination. Moreover, SEM observed rough surfaces of C_1_A_0.5_P_0.5_, C_1.5_A_0.5_P_0.5_, and C_2_A_0.5_P_0.5_. This rough surface occurred due to incomplete hydrogel formation [26], possibly because of insufficient water hyacinth cellulose in the formulations (Figure 6a–c). The C_2.5_A_0.5_P_0.5_ and C_3_A_0.5_P_0.5_, providing a complete hydrogel shape in the visual inspection, demonstrated a smooth surface (Figure 6d–e). Under a cross-sectional observation, all formulations showed numerous porosities with no significant differences, as illustrated in Figure 6f–i. Additionally, the porosity of the material might contribute to a high swelling ratio, which is related to the water absorption of formulations [27].

#### 2.4.3. Puncture Strength of Water Hyacinth Cellulose-Based Hydrogels

Puncture strength is used to reveal hydrogel robustness, since a high-puncture strength hydrogel tends to hold its shape during application better than a low-puncture strength hydrogel [28]. In addition, our findings show that the high amounts of cellulose in the formulation increased the puncture strength of the hydrogel, so C_3_A_0.5_P_0.5_, which has the highest amount of cellulose in the formulation, showed the highest puncture strength (2.76 ± 0.14 N/mm^2^). In contrast, C_1_A_0.5_P_0.5_, C_1.5_A_0.5_P_0.5_, and C_2_A_0.5_P_0.5_ showed the lowest puncture strength, with no significant difference (1.49 ± 0.13, 1.47 ± 0.12 and 1.53 ± 0.26 N/mm^2^) between each other (*p* < 0.05) (Table 2). The -OH groups of WHC in the formulations may form hydrogen bonds with alginate and pectin to raise puncture strength. In a previous study, the tensile strength of starch/WHC bioplastic increased with the addition of WHC [29]. In addition, another study found that increasing cellulose content had an important role in the support structure and led to enhancements in the mechanical strength of cellulose/sodium alginate hydrogels [25]. This result showed the same trend as our study.

#### 2.4.4. WHC-Composited Hydrogel Swelling

The swelling properties of hydrogels were evaluated by immersing cryogels in PBS pH 7.4 at 32 °C for 24 h. In addition, the swelling properties relate to hydrogel porosity and hydrophilicity as water passes through the polymeric structure and forms hydrogen bonds with hydrophilic polymers [30] in the formulation of water hyacinth cellulose, sodium alginate and pectin. However, in this study, the amounts of sodium alginate and pectin are equal, so the swelling ratio relied on the quantity of cellulose in each formulation. This study found that the swelling ratio tended to increase with the addition of WHC. In a previous study, the swelling of cellulose/PVA cryogels, which are freeze-dried hydrogels, increased with the increase in cellulose content in the formulation because the hydrophilicity of hydrogel increased, enhancing swelling when the amount of cellulose was increased [31]. The swelling ratio increased due to the hydrophilic nature of cellulose, presenting several -OH groups to form hydrogen with water molecules [32,33]. Although C_3_A_0.5_P_0.5_ had the highest amount of cellulose, it did not show the highest swelling ratio and water content because its structure was segregated during soaking in the PBS, which is related to low durability (Table 2). In this case, the cross-linked structure of pectin and alginate with Ca^2+^ had a limited ability to interact with WHC to form a hydrogel. Thus, the excess of WHC, which could not be accommodated by the pectin and alginate cross-linked structure, might be easily segregated from C_3_A_0.5_P_0.5_, leading to a lower swelling ratio and water content than C_2.5_A_0.5_P_0.5_. Thus, this study found that C_2.5_A_0.5_P_0.5_ possessed the significantly highest value for swelling ratio (173.28 ± 4.94%) and water content (71.93 ± 0.87%) (*p* < 0.05).

#### 2.4.5. Gel Content of WHC Hydrogels

The WHC-composited cryogel was cut and soaked in PBS pH 7.4 at 32 °C for 24 h, and the gel content was evaluated by means of the gravimetric method. Additionally, the lack of structural durability is one of the problems with hydrogels after contact with biological fluids or wound exudates. In other words, hydrogels with low durability typically segregate during application [34]. Our study found that the increase in the amount of WHC increased the gel content (Table 2). However, C_3_A_0.5_P_0.5_, which had the highest amount of cellulose, did not show the highest gel content because excess cellulose was added. Similar to in the case of swelling, the excessive WHC in C_3_A_0.5_P_0.5_ segregated from the hydrogel while being soaked in PBS. This led to the loss of mass in the formulation. Thus, the C_2.5_A_0.5_P_0.5_ was more durable than other WHC-composited hydrogels, demonstrating the significantly highest gel content (39.35 ± 0.53%). According to its visual appearance, morphological characteristics, puncture strength, swelling ratio, and gel content, the C_2.5_A_0.5_P_0.5_ was a suitable hydrogel formulation to be selected for loading quercetin as an active compound.

### 2.5. FTIR of WHC-Composited Hydrogels

**Figure 7:**
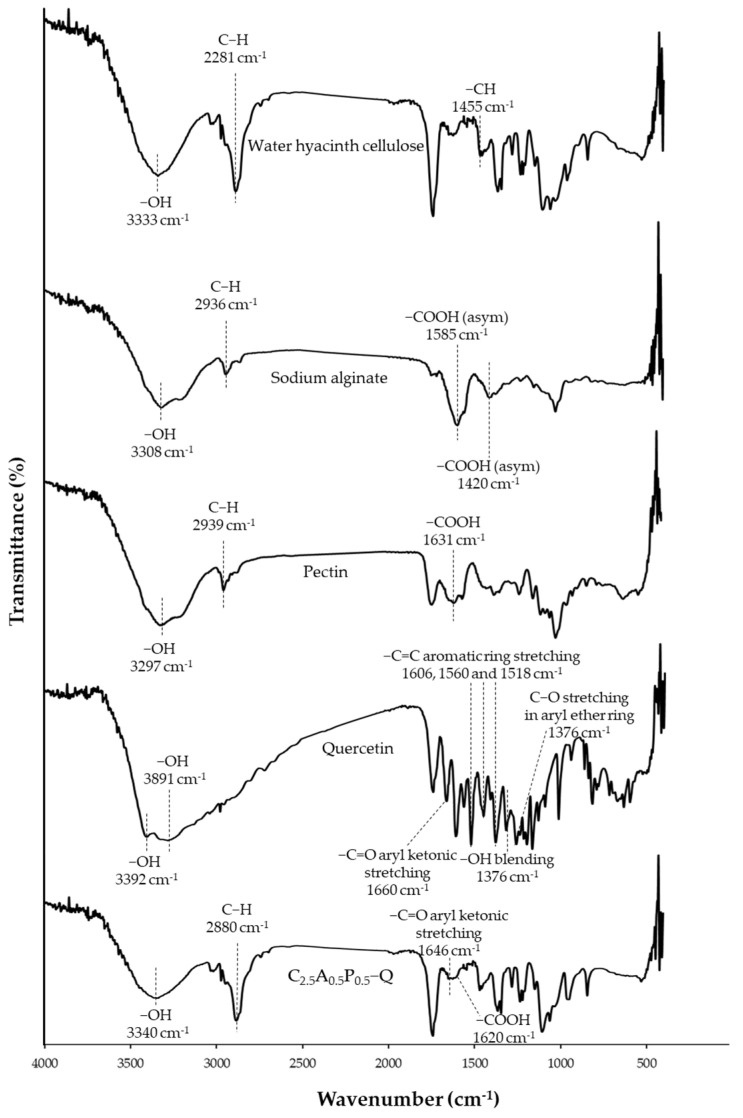
FTIR spectra of WHC-composited hydrogels.

FTIR examination indicated possible interactions in materials via chemical shifts or intensity changes, relating to chemical interactions among substances [35]. The FTIR spectra are shown in Figure 7. Additionally, the -OH peak change in the range 3170–3560 cm^−1^ may indicate hydrogen bond interactions in the hydrogel [36]. The crucial peaks of cellulose are -OH stretching and C-H stretching at 3333 and 2881 cm^−1^, respectively. In sodium alginate spectra, the wide band around 3308 cm^−1^ corresponds to -OH stretching. Moreover, the bands at 1585 and 1402 cm^−1^ relate to -COOH asymmetric and symmetric stretching, respectively [37,38]. The observed bands of pectin at 3297, 2939, and 1624 cm^−1^ correspond to -OH stretching, C-H stretching, and -COOH asymmetric stretching, respectively [39,40]. In quercetin spectra, the wide bands at 3392 and 3291 cm^−1^ correspond to -OH stretching. The sharp peak at 1660 cm^−1^ relates to C=O aryl ketonic stretching. The three peaks at 1606, 1560, and 1518 cm^−1^ correspond to C=C aromatic ring stretching, and the peak at 1250 cm^−1^ refers to C-O stretching in the aryl ether ring. Moreover, the peak band at 1376 cm^−1^ relates to -OH bending [35,41,42]. In the spectra of C_2.5_A_0.5_P_0.5_ containing quercetin (C_2.5_A_0.5_P_0.5_-Q), -COOH stretching of sodium alginate and pectin at 1585 and 1624 cm^−1^ shifted to 1620 cm^−1^, indicating the ionic interaction between Ca^2+^ and -COOH of sodium alginate and pectin during the crosslinking process. However, -COOH symmetric stretching of sodium alginate at 1402 cm^−1^ cannot be investigated because its peak overlapped with -CH stretching of WHC at 1455 cm^−1^. Moreover, the C_2.5_A_0.5_P_0.5_-Q spectra indicated that the OH peak changed to 3340 cm^−1^ in the region 3170–3560 cm^−1^, which showed relatively lower intensity. It may indicate hydrogen bonds between hydrophilic polymers—for instance, cellulose–sodium alginate, cellulose–pectin, and cellulose–quercetin [43,44]. Moreover, the peak of -OH bending of quercetin at 1376, shifting to 1363 cm^−1^ in C_2.5_A_0.5_P_0.5_-Q spectra, also possibly indicated hydrogen bonding with water hyacinth and/or other polymers in the formulation. Water hyacinth cellulose could form hydrogen bonding with quercetin. To clarify, several hydroxyl groups in quercetin molecules can form hydrogen bonds with oxygen atoms and hydroxyl groups in cellulose molecules [42,44]. Furthermore, the shift in the carbonyl peak of quercetin at 1660 cm^−1^ to a lower frequency at 1646 cm^−1^ in C_2.5_A_0.5_P_0.5_-Q also indicates the occurrence of hydrogen bonding [45]. The occurrence of a new peak of quercetin in the hydrogel was observed difficulty because several peaks of quercetin were overlapped by the peaks of cellulose, sodium alginate, and pectin. However, this study showed that the possible chemical interaction between water hyacinth cellulose and quercetin might be hydrogen bonding, formed by oxygen and hydrogen atoms in both molecules.

### 2.6. Quercetin-Loaded Content and Characteristics of WHC-Composited Hydrogels Containing Quercetin

The quercetin-loaded content in C_2.5_A_0.5_P_0.5_-Q formulation was 92.07 ± 5.77%. It meant that not only did the WHC-composited hydrogel have the potential to contain a bioactive compound, but also that quercetin was stable during the preparation of WHC-composited hydrogels. The formulation contained a high percentage of quercetin-loaded content because the water hyacinth cellulose could form hydrogen bonds with quercetin [42,44]. This bonding might enhance the ability of the formulation to contain quercetin. Furthermore, Jantarat et al. [44] found that the bacterial cellulose composite membranes showed approximately 98% quercetin-loaded content, indicating the potential of a cellulose material to load quercetin.

**Figure 8:**
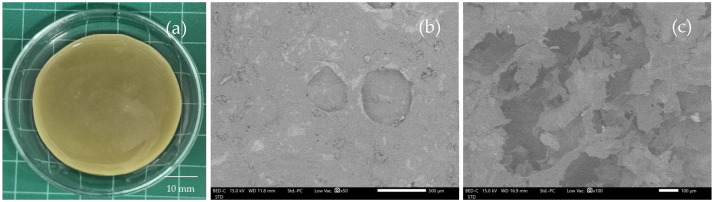
The visual appearance (**a**) and SEM micrograph of the surface and cross-section of C_2.5_A_0.5_P_0.5_-Q at 50× and 100× magnifications (**b**,**c**).

Upon visual inspection, the C_2.5_A_0.5_P_0.5_-Q was a regular shape with a yellow-green color due to the color of quercetin (Figure 8a). The thickness and diameter of C_2.5_A_0.5_P_0.5_-Q were 5.15 ± 0.17 and 44.33 ± 0.82 mm, respectively. The SEM micrograph showed a slightly rough surface of C_2.5_A_0.5_P_0.5_-Q (Figure 8b). In the cross-sectional observation, the C_2.5_A_0.5_P_0.5_-Q showed numerous porosities in the hydrogel’s matrix, similar to the hydrogel without quercetin (C_2.5_A_0.5_P_0.5_) (Figure 8c). Interestingly, the puncture strength significantly increased from 2.16 ± 0.14 to 2.50 ± 0.10 N/mm^2^ due to hydrogen bonding between WHC and quercetin, enhancing the hardness of the hydrogel (*p* < 0.05).

### 2.7. Cytotoxicity of WHC-Based Hydrogels

**Figure 9:**
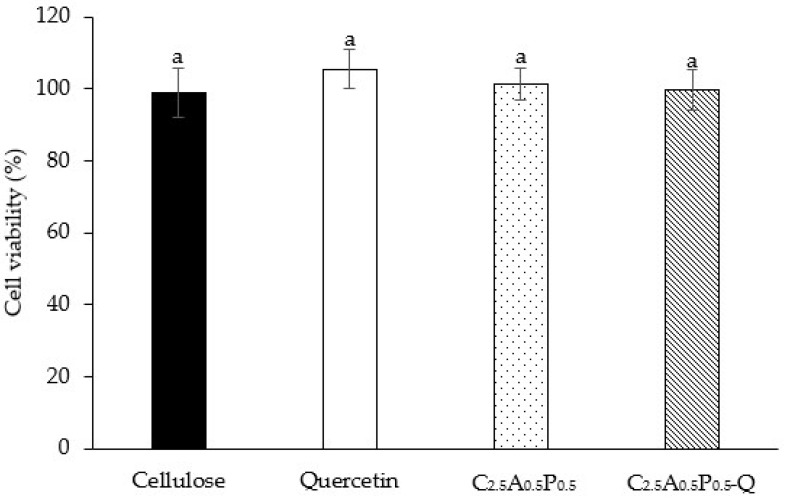
HaCaT cell viability after exposure to cellulose, quercetin, C_2.5_A_0.5_P_0.5_, and C_2.5_A_0.5_P_0.5_-Q. The results are demonstrated as mean ± S.D.; superscripts with the same letter (^a^) in each bar indicate an insignificant difference between samples (*p* > 0.05).

The cell viability of HaCaT cells, immortalized human skin keratinocytes, was investigated to ensure the safety of hydrogels on human cells. Moreover, the results of the MTT assay can be used to identify the optimal hydrogel formulation that can influence the cell viability of keratinocyte cells. The viability of HaCaT cells after being treated with cellulose, quercetin, C_2.5_A_0.5_P_0.5_, and C_2.5_A_0.5_P_0.5_-Q was 99.11 ± 6.87, 105.46 ± 5.41, 101.46 ± 4.51 and 99.72 ± 5.68%, respectively, as shown in Figure 9. In addition, there was no significant difference among hydrogel formulations. Thus, the C_2.5_A_0.5_P_0.5_-Q formulation is safe for human skin and suitable for topical drug delivery. In other words, C_2.5_A_0.5_P_0.5_-Q showed no toxicity to the human cell. Our previous study also found that the hydrogel composed of bacterial cellulose, alginate, and pectin did not possess cytotoxicity [43].

### 2.8. Antibacterial Activity of WHC-Based Hydrogels Containing Quercetin

In this study, the disk-diffusion method was applied to evaluate the antibacterial activity of C_2.5_A_0.5_P_0.5_-Q against *S. aureus* and *P. aeruginosa*. The antibacterial results are shown in Table 3. The C_2.5_A_0.5_P_0.5_ hydrogel without quercetin had no antibacterial activity against the tested bacteria. DMSO, used as the solvent for quercetin, also did not demonstrate antibacterial activity. Quercetin, used as the positive control, showed antibacterial activity against *S. aureus* and *P. aeruginosa* because generally, quercetin possesses antibacterial activity against the growth of both bacteria [46,47]. When quercetin was added, the zone of inhibition of C_2.5_A_0.5_P_0.5_-Q against *S. aureus* and *P. aeruginosa* significantly increased to 16.44 ± 1.06 and 21.01 ± 0.26 mm, respectively. The porous hydrogel structure increased the contact interface between loaded quercetin and bacteria to enhance antibacterial activity. In addition, the hydrogel matrix might entrap bacteria inside and thus prevent bacterial growth due to an unsuitable environment. Ye et al. found that a PEG hydrogel improved antibacterial activity due to increasing the contact interface and preventing bacterial growth [48].

## 3. Conclusions

Water hyacinth cellulose was isolated from the petiole of water hyacinth by means of alkaline hydrolysis and bleaching. The yield of the isolated product was reasonable and sufficient. The FTIR and XRD studies confirmed the efficacy of water hyacinth cellulose isolation. WHC-composited hydrogels were fabricated using sodium alginate and low methoxy pectin with CaCl_2_ as the crosslinking agent. Among five WHC-composited hydrogel formulations, C_2.5_A_0.5_P_0.5_ was selected to contain quercetin as an active compound for antibacterial activities. It demonstrates a regular shape, good mechanical properties, and the highest swelling ratio. The FTIR study showed signs of hydrogen bonding between WHC and quercetin. C_2.5_A_0.5_P_0.5_-Q was also successfully prepared and showed 92.07% of quercetin-loaded content. In an antibacterial experiment, C_2.5_A_0.5_P_0.5_-Q showed outstanding antibacterial activity against *S. aureus* and *P. aeruginosa* due to hydrogel properties. In addition, the cytotoxicity assay showed that C_2.5_A_0.5_P_0.5_-Q is safe for a human cell. Our study indicated that the cellulose-composited hydrogel from the WHC has the potential to be used as topical antibacterial material.

## 4. Materials and Methods

### 4.1. Materials

Calcium chloride (CaCl_2_) was purchased from RCI Labscan Ltd., Bangkok, Thailand. Low methoxy pectin (P; degree of esterification = 29%) was purchased from Cargill^TM^, Saint Germain, France. Polyethylene glycol 1500 (PEG; molecular weight 1500 g/mol) was purchased from Tinnakorn Chemical and Supply Co., Ltd., Bangkok, Thailand. Sodium alginate (A: molecular weight ~167 kDa, M/G = 65/35) was purchased from Qingdao Bright Moon Seaweed Group Co., Ltd., Qingdao, China. Deionized water (DI) was used as a solvent for preparing hydrogels.

### 4.2. Water Hyacinth Stems and Leaves Collection

Water hyacinth was collected from a natural river in Chiang Mai, Thailand, and washed with purified water. After the roots of water hyacinth were removed, stems and leaves were minced into small pieces, dried in a hot air oven at 50 °C for 24 h and reduced into powder by a grinding machine.

### 4.3. Cellulose Isolation

Water hyacinth powder was immersed in a toluene/ethanol solution with a ratio of 2:1 at 115 °C for 3 h to isolate water hyacinth cellulose. Moreover, the bleaching process was determined using 3% NaClO at 80 °C for 2 h. After that, the mixture was hydrolyzed using 1% NaOH at 60 °C for 2 h to eradicate hemicellulose. Then, 1% NaClO was applied to perform the second bleaching process and stirred continuously at 75 °C for 3 h. Consequently, the mixture was hydrolyzed in the final step using 5% HCl as a catalyst at 65 °C for 6 h. Additionally, the mixture was filtered and the acquired solid cellulose was washed with deionized water until a neutral pH was reached and finally dried in an oven (Memmert GmbH Co., KG, Schwabach, Germany) at 50 °C. At the end of each step, the remaining sample was washed several times with deionized water. The extraction yield was calculated according to the initial weight of water hyacinth power and the final dried solid cellulose using Equation (1).

(1) $$\mathrm{Extraction}\;\mathrm{yield}\;(\%)=\frac{\mathrm{The}\;\mathrm{final}\;\mathrm{dried}\;\mathrm{solid}\;\mathrm{cellulose}}{\mathrm{The}\;\mathrm{initial}\;\mathrm{weight}\;\mathrm{of}\;\mathrm{water}\;\mathrm{hyacinth}\;\mathrm{powder}\;}\;\times \;100$$

### 4.4. Cellulose Identification

The identification of extracted cellulose and raw material was investigated by Fourier transform infrared (FTIR) spectrometer (FT/IR-4700, Jasco, Tokyo, Japan). The tested samples were dried in a hot air oven at 60 °C for 4 h, or until the moisture content of the samples was less than 5%. The moisture content was measured by a moisture analyzer (HC103, Mettler Toledo, Columbus, OH, USA) at 105 °C of drying temperature. All samples were scanned at 4 cm^−1^ resolution in transmittance mode from 400 to 4000 cm^−1^.

### 4.5. X-rays Diffractometry (XRD)

Cellulose and raw materials were evaluated regarding their crystalline state by preparing their liquisolid compacts and using the analytical X-ray diffractometer (Miniflex ll, Rigaku corporation, Tokyo, Japan) with the conditions of 40 kV voltage rate, 0.4 s/step counting rate, and a scanning range from 5 to 70° to evaluate thte crystalline state.

### 4.6. Preparation of Water Hyacinth Cellulose-Composited Hydrogels

Water hyacinth cellulose (WHC)-composited hydrogels were prepared by dispersing WHC in 50 g of 40% *w*/*w* polyethylene glycol 1500 (PEG 1500) using the homogenizer (IKA T25 Ultra-Trurrax, IKA laboratory technology, Staufen, Germany) at 3500 rpm for 30 min, and dissolving sodium alginate and pectin in deionized water to obtain the polymeric solution. Then, the cellulose suspension was mixed with the polymer solution at different ratios using the homogenizer (Table 4). The quercetin was loaded into the polymeric mixture in a concentration of 0.1% *w*/*w* based on the total weight of the formulation or 0.1 g in 100 g of the formulation. One milliliter of the quercetin solution at 0.1 g/mL in dimethyl sulfoxide (DMSO) was mixed homogeneously with the polymeric mixture by the homogenizer. The weight was adjusted to 100 g with deionized water. Then, 10 g of the mixture was poured into a Petri dish and crosslinked to form a hydrogel by gradually adding 3% *w*/*v* calcium chloride solution (CaCl_2_) at room temperature for 2 h. After that, the hydrogels were washed several times with deionized water to eliminate excess calcium ions. Finally, the hydrogels were dried using a freeze-drying machine (Christ Beta 2-8 LD-plus, Osterode am Harz, Germany) for 24 h and kept in an air-tight container.

### 4.7. Physicochemical Property Identification of WHC-Composited Hydrogels

#### 4.7.1. Hydrogel Appearance Evaluation

The obtained hydrogel from the cross-linking process with Ca^2+^ was evaluated for its physical appearance by means of visual inspection. The complete hydrogel formation should not have pores, holes, collapses on the hydrogel surface, or broken hydrogel pieces. The thickness and diameter of hydrogels were measured by an outside micrometer (3203-25A, Insize Co, Ltd., Suzhou New District, Jiangsu, China). The standard deviation of the thickness of the hydrogels was lower than 0.5 mm to present a smooth surface. The diameter of hydrogels was used to evaluate the size of the hydrogel.

#### 4.7.2. Morphological Characterization

All freeze-dried hydrogel (cryogel) samples were cut and fixed on an aluminum stub with double-sided adhesive carbon tape. After coating with gold for 1 min, the surface and cross-sectional images of the specimens were investigated at 50× and 100× magnification levels using scanning electron microscopy (SEM) (JEOL JCM-7000 NeoScope™ Benchtop, Tokyo, Japan) at 15 kV under low vacuum mode to evaluate internal surface and porosity. Moreover, the measurements of hydrogel thickness and diameter were performed in triplicate for each sample using a micrometer (3203-25A, Insize Co, Ltd., Suzhou, China).

#### 4.7.3. Mechanical Properties

The elasticity of hydrogels was investigated by a texture analyzer, TX. TA plus (Stable Micro Systems, Surrey, UK) with load cell 5 kg (0.001 N of sensitivity) in the compression mode. The heavy-duty platform and plane flat-faced surface probe with a 2 mm-diameter cylindrical stainless probe were used to perform the test. The probe speed was set at 2.00 mm/s. The experiment was performed at room temperature. Each experiment was repeated 5 times. Puncture strength (N/mm^2^) was calculated by the following Equation (2) [49].

(2) $$\mathrm{Puncture}\;\mathrm{strength}=\frac{Fmax}{A}$$

where *Fmax* is the force at the breaking point (N) and *A* is the surface area in contact with the probe surface (mm^2^).

#### 4.7.4. Swelling Ratio and Water Content

Cryogels were cut into 2 × 2 cm^2^ pieces and weighted by an analytical balance before immersion in phosphate buffer saline (PBS) pH 7.4 at 32 °C. The samples were weighed again after 24 h of immersion. Before weighing with the analytical balance, the hydrogel was blotted with a filter paper to remove excess PBS. Then, the swelling ratio was calculated using Equations (3) and (4), respectively.

(3) $$\mathrm{Swelling}\;\mathrm{ratio}\;(\%)=\frac{W_{t}-W_{0}}{W_{0}}\;\times \;100$$

where *W*_0_ is the initial weight of a sample (g) and *W_t_* is the weight of a sample (g) at 24 h.

#### 4.7.5. Gel Content

Cryogels were cut into 2 × 2 cm^2^ pieces and weighed using an analytical balance before immersion in PBS pH 7.4 at 32 °C. After 24 h of immersion tine, the samples were removed from the PBS, blotted with filter paper to removed excess PBS, and dried in an oven (Memmert GmbH Co., KG, Schwabach, Germany) at 70 °C and weighed with an analytical balance. Moreover, each sample was tested in triplicate. Gel content was calculated using Equation (4) [50,51,52].

(4) $$\mathrm{Gel}\;\mathrm{Content}\;(\%)=\;\frac{W_{i}}{W_{f}}\;\times \;100$$

where *W_i_* is an initial weight (g) of a sample and *W_f_* is a weight of a sample after immersion for 24 h (g).

### 4.8. FTIR of WHC-Composited Hydrogels

Chemical interactions of hydrogel components were investigated by a Fourier transform infrared (FTIR) spectrometer (FT/IR-4700, Jasco, Tokyo, Japan). The cryogel and material samples were dried in a hot air oven at 60 °C until the moisture content of the samples was less than 5%. The moisture content was measured by a moisture analyzer with 105 °C drying temperature. All the samples were scanned at 4 cm^−1^ resolution in transmittance mode from 400 to 4000 cm^−1^.

### 4.9. Quercetin Loading Efficiency

The analysis method was adapted from previous studies [43,53]. One hundred milligrams (100 mg) of WHC-composited cryogel containing quercetin was mixed in 10 mL of dimethyl sulfoxide (DMSO) with a magnetic stirrer for 1 h. The hydrogel structure was destroyed using a sonicator (Elmasonic S100H, Elma, Singen, Germany) for 1 h. After that, the suspension was centrifuged by a centrifuge (MPW-352R, Warsaw, Poland) at 12,000 rpm for 20 min to separate sediment and supernatant. Then, the obtained supernatant was diluted with ethanol:water (1:1) and filtered with a 0.45 μm membrane before analysis with a UV-spectrophotometer (UV2600i, Shimadzu Corporation, Kyoto, Japan) at 372 nm. Additionally, the amount of quercetin in the cryogel was calculated from the quercetin standard curve with high linear regression (*r*^2^ = 0.999). Moreover, the sedimented hydrogel was also evaluated for remaining quercetin in the sample by the same method to ensure that no quercetin was in the sediment. In other words, the quercetin was completely released through DMSO. The quercetin loading efficiency was calculated by Equation (5) [54].

(5) $$\mathrm{Quercetin}\;\mathrm{loading}\;\mathrm{efficiency}=\;\frac{\mathrm{The}\;\mathrm{amount}\;\mathrm{of}\;\mathrm{quercetin}\;\mathrm{in}\;\mathrm{hydrogel}}{\mathrm{Theoretical}\;\mathrm{quercetin}\;\mathrm{content}\;\mathrm{in}\;a\;\mathrm{formualtion}}\;\times \;100$$

### 4.10. Antimicrobial Test

The antibacterial activity test was performed similarly to a previous study [43]. Stock cultures of *Staphylococcus aureus* (ATCC25923) and *Pseudomonas aeruginosa* (ATCC27853) were grown in brain heart infusion (BHI) medium (HiMedia, Mumbai, India) at 37 °C for 24 h. The cultures were subsequently inoculated into tryptic soy broth (Sigma-Aldrich, St. Louis, MO, USA), and grown at 37 °C under aerobic atmosphere for 12 h. The optical density of overnight cultures of *S. aureus* and *P. aeruginosa* was measured at 600 nm wavelength (OD600) using a spectrophotometer (Beckman Coulter, Fullerton, CA, USA) before the fresh preparation of bacterial stocks.

Before the antibacterial test, all tested WHC-composited cryogels were sterilized by ethylene oxide. The agar disk-diffusion method was used to evaluate the hydrogel’s ability to kill two tested skin pathogens. Briefly, 100 µL of bacterial stock (OD600 = 0.1) was plated on a dried agar plate and allowed to dry. Whatman^®^ antibiotic assay discs (GE Healthcare, Pittsburgh, PA, USA) with 0.4 mm of diameter were loaded with 20 µg of 30 mg/mL quercetin and then placed on the prepared agar plate and left in a 37 °C aerobic incubator for 24 h. To evaluate the antibacterial activity of the hydrogel containing quercetin with the agar disk-diffusion method, the blank WHC-composited cryogel and the quercetin solution in DMSO were used as negative and positive controls, respectively. Finally, antibacterial activity was evaluated by the measurement of the zone of inhibition’s diameter using a Mitutoyo^®^ Digimatic caliper (Mitutoyo Corporation, Kanagawa, Japan). Three independent experiments in triplicate were performed.

### 4.11. Cell Culture and Cell Viability Assay

The method to analyze cell viability with MTT assay was described in a previous study [43]. HaCaT, a human keratinocyte cell line, was cultured in Dulbecco’s modified Eagle’s medium (DMEM) supplemented with both 1% penicillin-streptomycin and 10% fetal bovine serum (FBS). Then, it was incubated at 37 °C and 5% CO_2_ in a humidified incubator. The selected sample was analyzed in a cell viability assay using 3-(4,5-dimethylthiazol-2-yl)-2,5-diphenyl-tetrazolium bromide (MTT) (Sigma-Aldrich, Saint Louis, MO, USA). HaCaT cells were seeded in 96-well plates at a density of 8 × 10^3^ cells per well and incubated for 24 h in a culture medium. The selected WHC-based cryogel samples (C_2.5_A_0.5_P_0.5_ and C_2.5_A_0.5_P_0.5_-Q) (2 × 2 × 2 mm^3^), WHC powder, and 0.1% *w*/*v* quercetin solution in DMSO were soaked in DMEM for 48 h. The extracted solution was filtered through a 0.22 μm membrane filter and incubated with HaCaT cells at 37 °C with 5% CO_2_. After 24 h of incubation, samples were removed. Then, 0.5 mg/mL MTT was added (100 µL/well) and incubated at 37 °C for 2 h. Subsequently, the medium was replaced with DMSO (100 µL) to solubilize the formazan product. After that, the samples were measured for their absorbance at 550 nm using a microplate reader (Spectramax M3, Molecular Devices, San Jose, CA, USA). The percentage of cell viability was calculated by Equation (6).

(6) $$\mathrm{Cell}\;\mathrm{viability}\;(\%)=\frac{A550\;\mathrm{of}\;\mathrm{treatment}}{A550\;\mathrm{of}\;\mathrm{control}}\;\times \;100$$

where A550 is the absorbance at 550 nm.

### 4.12. Statistical Analysis

This study employed the one-way ANOVA test to evaluate the significant difference when the *p*-value was less than 0.05 using the SPSS software version 17.0 (IBM Corporation, Armonk, NY, USA), and the results are shown as the means ± standard deviations (S.D.).

## Figures and Tables

**Table 1:** The thickness and diameter of WHC-hydrogels.

Sample	Thickness (mm)	Diameter (mm)
C_1_A_0.5_P_0.5_	4.05 ± 0.71 ^a^	40.63 ± 3.13 ^a^
C_1.5_A_0.5_P_0.5_	4.03 ± 0.79 ^a^	42.94 ± 1.61 ^ab^
C_2_A_0.5_P_0.5_	4.37 ± 0.67 ^b^	42.16 ± 1.47 ^ab^
C_2.5_A_0.5_P_0.5_	5.29 ± 0.15 ^c^	44.75 ± 1.11 ^b^
C_3_A_0.5_P_0.5_	5.71 ± 0.23 ^c^	44.43 ± 1.25 ^b^

The results are demonstrated as Mean ± S.D. Different letters of superscripts (^a, b, c^) in the same column indicate a significant difference between the samples (*p* < 0.05).

**Table 2:** Puncture strength, swelling, and gel content of hydrogel.

Formulations	Puncture Strength (N/mm^2^)	Swelling Ratio (%)	Gel Content (%)
C_1_A_0.5_P_0.5_	1.49 ± 0.13 ^a^	102.95 ± 14.96 ^a^	20.48 ± 1.64 ^a^
C_1.5_A_0.5_P_0.5_	1.47 ± 0.12 ^a^	109.94 ± 11.98 ^a^	21.39 ± 1.14 ^a^
C_2_A_0.5_P_0.5_	1.53 ± 0.26 ^a^	132.82 ± 9.41 ^b^	19.16 ± 1.82 ^a^
C_2.5_A_0.5_P_0.5_	2.16 ± 0.14 ^b^	173.28 ± 4.94 ^c^	39.35 ± 0.53 ^b^
C_3_A_0.5_P_0.5_	2.76 ± 0.14 ^c^	118.64 ± 3.22 ^a^	20.56 ± 1.23 ^a^

The results are demonstrated as Mean ± S.D. Different letters of superscripts (^a, b, c^) in the same column indicate a significant difference between the formulations (*p* < 0.05).

**Table 3:** Anti-bacterial activity of the WHC-composited hydrogel against *S. aureus* and *P. aeruginosa*.

Samples	Zone of Inhibition (mm)	
	*S. aureus*	*P. aeruginosa*
C_2.5_A_0.5_P_0.5_	ND	ND
C_2.5_A_0.5_P_0.5_-Q	16.44 ± 1.06 ^a^	21.01 ± 0.26 ^a^
Quercetin (30 mg/mL)	10.56 ± 1.02 ^b^	13.08 ± 0.71 ^b^
DMSO	ND	ND

The results are demonstrated as Mean ± S.D. Different letters of superscripts (^a, b^) in the same column indicate a significant difference between the samples (*p* < 0.05).

**Table 4:** Polymer compositions of water hyacinth cellulose-composited hydrogels.

Sample Code	Polymer Composition (% *w*/*w*)		
	Water Hyacinth Cellulose (C)	Alginate (A)	Pectin (P)
C_1_A_0.5_P_0.5_	1.0	0.5	0.5
C_1.5_A_0.5_P_0.5_	1.5	0.5	0.5
C_2_A_0.5_P_0.5_	2.0	0.5	0.5
C_2.5_A_0.5_P_0.5_	2.5	0.5	0.5
C_3_A_0.5_P_0.5_	3.0	0.5	0.5

## Data Availability

Not applicable.
